# Digital biomarkers from geolocation data in bipolar disorder and schizophrenia: a systematic review

**DOI:** 10.1093/jamia/ocz043

**Published:** 2019-04-26

**Authors:** Paolo Fraccaro, Anna Beukenhorst, Matthew Sperrin, Simon Harper, Jasper Palmier-Claus, Shôn Lewis, Sabine N Van der Veer, Niels Peek

**Affiliations:** 1 Centre for Health Informatics, Division of Informatics, Imaging and Data Sciences, University of Manchester, Manchester, United Kingdom; 2 Hartree Centre STFC Laboratory, IBM Research UK, Warrington, United Kingdom; 3 Centre for Epidemiology, Division of Musculoskeletal & Dermatological Sciences, University of Manchester, Manchester, United Kingdom; 4 School of Computer Science, University of Manchester, Manchester, United Kingdom; 5 Division of Psychology & Mental Health, University of Manchester, Manchester, United Kingdom; 6 Greater Manchester Mental Health NHS Foundation Trust, Manchester, United Kingdom; 7 National Institute of Health Research Greater Manchester Patient Safety Translational Research Centre, University of Manchester, Manchester, United Kingdom; 8 National Institute of Health Research Manchester Biomedical Research Centre, Manchester Academic Health Science Centre, University of Manchester, Manchester, United Kingdom

**Keywords:** schizophrenia, bipolar disorder, serious mental illness, smartphone, geographical positioning system, geolocation

## Abstract

**Objective:**

The study sought to explore to what extent geolocation data has been used to study serious mental illness (SMI). SMIs such as bipolar disorder and schizophrenia are characterized by fluctuating symptoms and sudden relapse. Currently, monitoring of people with an SMI is largely done through face-to-face visits. Smartphone-based geolocation sensors create opportunities for continuous monitoring and early intervention.

**Materials and Methods:**

We searched MEDLINE, PsycINFO, and Scopus by combining terms related to geolocation and smartphones with SMI concepts. Study selection and data extraction were done in duplicate.

**Results:**

Eighteen publications describing 16 studies were included in our review. Eleven studies focused on bipolar disorder. Common geolocation-derived digital biomarkers were number of locations visited (n = 8), distance traveled (n = 8), time spent at prespecified locations (n = 7), and number of changes in GSM (Global System for Mobile communications) cell (n = 4). Twelve of 14 publications evaluating clinical aspects found an association between geolocation-derived digital biomarker and SMI concepts, especially mood. Geolocation-derived digital biomarkers were more strongly associated with SMI concepts than other information (eg, accelerometer data, smartphone activity, self-reported symptoms). However, small sample sizes and short follow-up warrant cautious interpretation of these findings: of all included studies, 7 had a sample of fewer than 10 patients and 11 had a duration shorter than 12 weeks.

**Conclusions:**

The growing body of evidence for the association between SMI concepts and geolocation-derived digital biomarkers shows potential for this instrument to be used for continuous monitoring of patients in their everyday lives, but there is a need for larger studies with longer follow-up times.

## INTRODUCTION

Adoption of personal digital devices continues to grow, with an estimated 3 billion people currently using smartphones across the globe.^1^ As ownership and adoption increases in patients living with mental health conditions,^2–4^ there arise new opportunities for symptom monitoring and interventions in mental health care.^2^^,^^5–8^ In particular, the ubiquitous presence of smartphones might allow a higher number of patients to access care, independently from their location,^9^ offsetting the shortage of staff and resources in mental health worldwide.^7^^,^^10^ Furthermore, smartphones provide unprecedented opportunities to introduce objectivity into assessment, treatment and monitoring of mental health conditions.^7^^,^^9^^,^^11^ Embedded sensors on smartphones can capture rich information on proxy indicators of behavior and personal experience in daily life through “passive” data collection (ie, using routinely collected data from embedded sensors and interactions of users with their smartphone).^12^ This approach toward measuring and characterising mental health conditions is called digital phenotyping.^13^

Digital phenotyping is especially relevant in areas such as serious mental illness (SMI) where conditions (ie, bipolar disorder and schizophrenia in the context of this review), due to the high risk of relapse,^14^^,^^15^ require close monitoring for long periods of time.^14^^,^^16^ Current monitoring methods rely on regular face-to-face visits, either in clinic, in the community or in people’s home.^17^ During these visits, healthcare professionals need to capture the dynamic expressions of SMI, which are often characterized by different combinations of fluctuating symptoms.^14^^,^^15^ Such symptoms affect mood, level of activity, and life regularity, as well as social functioning, which considers different aspects of at-home and out-of-home behaviors (eg, independence, withdrawal, and social and recreational activities).^14^^,^^18–20^ Digital phenotyping has the potential to measure and characterize these aspects of SMI more continuously. In turn, this may support better tailoring the frequency of visits to patients’ needs, and more timely interventions in case of an anticipated relapse.^21^

Faurholt-Jepsen et al^22^ recently advocated the potential of smartphone-collected data to study bipolar disorder. They suggested that illness status might be associated with patients’ social interactions (eg, number of texts, conversations, and number and duration of calls) and mobility derived from geolocation data. The latter, nowadays easily captured with sensors embedded in smartphones (eg, Global Positioning System [GPS] trackers and cellular network), is particularly interesting in SMI, as it also has the potential to continuously and passively measure other relevant aspects of behavior, such as life regularity and out-of-home activities. Geolocation data are already used similarly in other nonhealthcare applications: navigation systems to guide users in their journeys^23^; location-based recommender systems, which suggest to the user nearby places based on his or her previous location history and preferences^24^^–27^; social networking services that, based on users’ previous locations history, connect individuals with similar interests^28–30^; and monitoring of criminal offenders based on their location.^31^

However, there are concerns that continuously monitoring people’s location might induce paranoia in patients living with SMI,^21^ as well as raise ethical and privacy concerns.^32^ This happens despite the fact that inferring actual users behaviors from geolocation data is not straightforward at all. There is no clear indication of which geolocation-derived digital biomarkers are more appropriate to characterize and monitor behaviors or how to derive them. Furthermore, quality and quantity of collected geolocation data can be an issue.^33^ Geolocation data are subject to measurement error, which has currently a median of 70 m.^34^ In addition, geolocation data are prone to missing values.^35^ This often happens due to signal loss when entering buildings,^26^ users not carrying their device with them,^35^ and battery draining.^36–38^ Even after noise is removed, raw geolocation data (eg, spatiotemporal locations of an individual) must be carefully processed to extract any meaningful information. If more than quantitative statements such as “more activity” and “less activity” is needed, geolocation data trajectories must be enriched with geographic and semantic information from databases such as Google Maps,^39^ OpenStreetMap,^40^ and Foursquare.^30^ These often allow users to contribute information and therefore may be inaccurate or incomplete.^41^

In this systematic review of the literature, we aimed to evaluate the current state of the art in using geolocation data to assess SMI phenotypes. Our objectives were to identify what geolocation-derived digital biomarkers were used in SMI studies, how they were calculated, and to what extent they were associated with clinically relevant concepts.

## MATERIALS AND METHODS

We followed the PRISMA (Preferred Reporting Items for Systematic Reviews and Meta-Analyses) statement^42^ to design and report our systematic review, where applicable.

### Search strategy

In compliance with guidance from the Cochrane collaboration,^43^ we searched MEDLINE^44^ and PsycINFO^45^ via Ovid and Scopus^46^ for articles in English using words in title, abstract, or keywords, as well as standardized indexing terms. We combined terms referring to geolocation measurement and smartphones with SMI-related terms (ie, bipolar disorder and schizophrenia). Supplementary file A contains the search syntax implemented for each database. The searches were performed on March 8, 2018. No limits were applied to the year of publication.

#### Selection of relevant studies

In our systematic review, we were interested in studies adhering to the following criteria:

- Original articles in English, as well as articles published in conference proceedings. Systematic reviews were included in the full text-analysis for reference checking, but excluded from the actual review. We also excluded publications describing a system but without having tested it, conference abstracts, narrative reviews, editorials, viewpoint papers, and gray literature;
- Studies reporting on systems that had patients with bipolar disorder and schizophrenia as the target users, while excluding studies on other mental health conditions. We also included studies in healthy volunteers as long as the system was designed for ultimate use in patients with SMI;
- Studies on any type of system that measured geolocation, including smartphones or other dedicated sensors. Geolocation could be measured via: GPS; GSM (Global System for Mobile communications) cellular network, by looking at cell tower IDs; WiFi, by considering to which WiFi network a device is connected (eg, clinic or home); and Bluetooth, by considering the known position of devices near the user.

Our aim was to get a comprehensive overview of relevant studies, and therefore we did not exclude studies based on study design, study quality, or sample size. After removing duplicates from the MEDLINE, PsycINFO, and Scopus searches, 2 reviewers (PF and AB) independently screened all titles and abstracts. For potentially relevant studies, we retrieved the full text; these were assessed for relevance by the same reviewers (PF and AB). Disagreement was resolved through discussion at each stage.

### Data extraction and synthesis

We developed a data abstraction form, and pilot-tested it, for clarity and comprehensiveness, among the authors (PF, AB, SNVdV, NP). The final form included study characteristics, target population (bipolar disorder, schizophrenia), technology used to measure geolocation (GPS, WiFi, Bluetooth, or GSM cellular network), sample frequency, methods to process geolocation data, geolocation-derived digital biomarkers, findings (ie, in terms of association with clinically relevant concepts), and data collected in addition to geolocation. Two authors (PF and AB), independently and in duplicate, extracted all data from the included studies. Disagreement was resolved via discussion.

We performed a narrative synthesis of the extracted data by focusing on 3 main areas: study characteristics, technological and methodological aspects, and association with clinically relevant concepts.

## RESULTS

**Figure 1.**
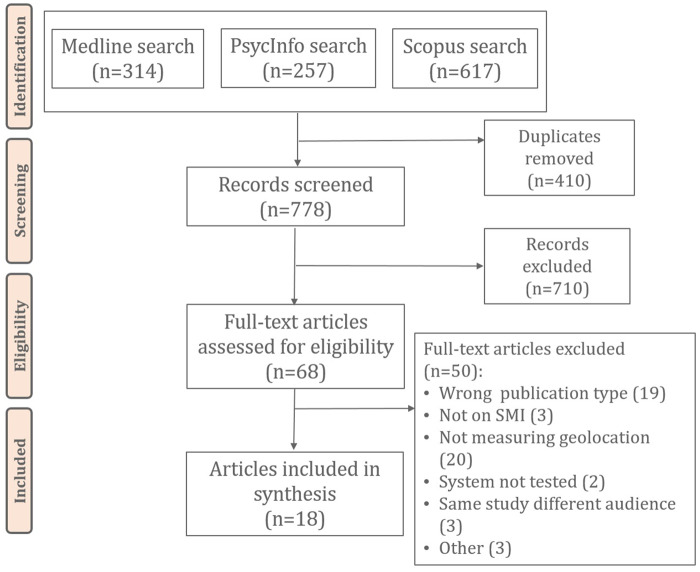
Flow diagram of screening and inclusion of relevant articles. SMI: serious mental illness.

Figure 1 shows the results from our review process. The searches yielded a total of 778 unique records included in the title and abstract screening. Of these, 68 were included in the full-text analysis, with 50 excluded according to our exclusion criteria. Among those excluded were studies^47–49^ that reported on 1 of the other included studies, but for a different audience. We also excluded 1 study^50^ because although their system collected geolocation data, they did not report on this in the results. Eighteen articles were finally included in the review.^51–68^

### Study characteristics

The included articles described 16 unique studies, with 3 publications reporting preliminary,^66^ final,^67^ and additional analyses^68^ for the same experiment. The vast majority of the included studies were pilot studies (ie, assessing feasibility and acceptability of the passive monitoring approach) or cohort studies; 2 studies^59^^,^^63^ presented data from a randomized controlled trial and a case-control study, respectively. Studies could be arranged into 9 distinct projects; 4 came from the MONARCA (MONitoring, treAtment and pRediCtion of bipolAr Disorder Episodes) collaborative: a European Union-funded initiative that aimed to monitor behavior via smartphone-collected data.^53–55^^,^^67^ The other main group of studies was represented by the ones using the CrossCheck app for schizophrenia (n = 4).^62–65^

Most of the 16 unique studies were published after 2013 (n = 13) and were conducted in Europe. No studies were published before 2010. Bipolar disorder was the most commonly targeted diagnosis (n = 11). The majority of studies included 20 or fewer patients (n = 12). All studies were prospective, with only 5 that lasted longer than 12 weeks (Table 1).

**Table 1.** General characteristics of included unique studies (n = 16)

General Project Characteristics	Data	Reference(s)
Year of publication		
2010-2013	3 (19)	^51–53^
2014-2016	9 (56)	^54–58^ ^,^ ^61–63^ ^,^ ^67^
After 2016	4 (25)	^59^ ^,^ ^60^ ^,^ ^64^ ^,^ ^65^
Geographical location^a^		
Europe	9 (67)	^51–55^ ^,^ ^57–59^ ^,^ ^67^
United States	7 (33)	^56^ ^,^ ^60–65^
Other	1 (8)	^61^
Target population^a^		
Bipolar disorder	11 (75)	^51–56^ ^,^ ^58^ ^,^ ^59^ ^,^ ^61^ ^,^ ^64^ ^,^ ^67^
Schizophrenia	6 (25)	^57^ ^,^ ^60^ ^,^ ^62–65^
Sample size		
<10	7 (44)	^51–53^ ^,^ ^56^ ^,^ ^57^ ^,^ ^61^ ^,^ ^65^
10-20	5 (31)	^54^ ^,^ ^58^ ^,^ ^60^ ^,^ ^62^ ^,^ ^67^
More than 20	4 (25)	^55^ ^,^ ^59^ ^,^ ^63^ ^,^ ^64^
Study length		
<6 wk	7 (44)	^51^ ^,^ ^52^ ^,^ ^56^ ^,^ ^57^ ^,^ ^61^ ^,^ ^62^ ^,^ ^64^
6-12 wk	4 (25)	^54^ ^,^ ^55^ ^,^ ^60^ ^,^ ^67^
>12 wk	5 (31)	^53^ ^,^ ^58^ ^,^ ^59^ ^,^ ^64^ ^,^ ^65^

Values are n (%).

a Categories are not mutually exclusive.

**Table 2.** Technological and methodological characteristics of included publications (n = 18)

Evaluation Characteristics	Data	Reference(s)
Technology to measure geolocation		
GPS	5 (28)	^60^ ^,^ ^61^ ^,^ ^63^ ^,^ ^66^ ^,^ ^67^
GPS + Bluetooth	2 (11)	^51^ ^,^ ^52^
GPS + GSM Cellular Network	1 (6)	^58^
GPS + GSM Cellular Network + WiFi	4 (22)	^56^ ^,^ ^57^ ^,^ ^59^ ^,^ ^65^
GPS + GSM Cellular Network + WiFi + Bluetooth	1 (6)	^62^
GSM Cellular Network	3 (17)	^53–55^
WiFi	1 (6)	^68^
Bluetooth	1 (6)	^64^
Sample frequency		
Time scale	10 (56)	^51–56^ ^,^ ^59–61^ ^,^ ^65^
Space scale	1 (6)	^57^
Not reported	7 (39)	^58^ ^,^ ^62–64^ ^,^ ^66–68^
Method to process geolocation data reported?		
Yes	11 (61)	^51^ ^,^ ^52^ ^,^ ^56^ ^,^ ^57^ ^,^ ^59^ ^,^ ^60^ ^,^ ^63^ ^,^ ^64^ ^,^ ^66–68^
No	7 (39)	^53–55^ ^,^ ^58^ ^,^ ^61^ ^,^ ^62^ ^,^ ^65^
Additional data to geolocation collected?		
Yes	16 (89)	^53–68^
No	2 (11)	^51^ ^,^ ^52^

Values are n (%).

GPS: global positioning system; GSM: Global System for Mobile communications.

### Technological and methodological characteristics

Table 2 shows the technological and methodological characteristics of included articles. The number of publications measuring geolocation with a single technology was 10,^53–55^^,^^60^^,^^61^^,^^63^^,^^64^^,^^66–68^ with 8 measuring it with multiple technologies.^51^^,^^52^^,^^56–59^^,^^62^^,^^65^ Overall the most common tool to measure geolocation was GPS embedded in participants' smartphone (n = 13),^51^^,^^52^^,^^56–63^^,^^65–67^ followed by GSM cellular network (n = 9)^53–59^^,^^62^^,^^65^ and WiFi (n = 6).^56^^,^^57^^,^^59^^,^^62^^,^^65^^,^^68^ Ten publications reported the geolocation data sample frequency, which was often at least once every 5 minutes.^51–55^^,^^61^ One publication adopted a space scale as the sample frequency; they recorded geolocation whether there was a change in position of at least 10 m. Although many of the included articles mentioned geolocation data quality as a potential issue,^52^^,^^57^^,^^59^^,^^60^^,^^63^^,^^66^^,^^67^ only 2 reported the actual amount of data collected during the study, such as 75%^65^ and 78.2%,^58^ respectively. Only 2 publications mentioned data imputation for missing geolocation data points.^59^^,^^60^

In terms of geolocation data processing, 11 publications clearly reported the method used to process the geolocation data. Five publications^51^^,^^52^^,^^56^^,^^63^^,^^68^ applied Ester et al,^69^ which is a density-based algorithm similar to the K-means method that allows clustering of geolocation data points to find meaningful locations.

Finally, the vast majority of included publications collected information in addition to the geolocation data: self-reported mood or symptoms (n = 13)^53–55^^,^^58–61^^,^^63–68^; additional smartphone-sensed data (eg, accelerometer, audio, calls, texts and smartphone activities; n = 13)^53–56^^,^^58^^,^^60^^,^^62–68^; and clinical assessment (n = 8), which was performed by clinicians against validated mania and depression rating scales,^54^^,^^55^^,^^58^^,^^65–67^ such as the Hamilton Depression Rating Scale [HDRS],^70^ Young Mania Rating Scale [YMRS],^71^ Quick Inventory of Depressive Symptomatology–Self-Report,^72^ and Self-Rating Mania Scale^73^; or reviewing patients’ electronic health records to identify events of interest.^60^^,^^65^

### Geolocation-derived digital biomarkers

Various digital-biomarkers were derived from raw geolocation data (see Table 3). They can be divided in 4 main groups. The first group aims at assessing mobility. This type of approach completely disregards the meaning associated with specific places on the map, and only considers patterns of movements derivable from geolocation data. Distance traveled was the most derived digital biomarker (n = 8), with number of changes in GSM cell ids (n = 4) being the most used in case only the GSM cellular network was used to measure geolocation. The second group of digital biomarkers focused on deriving information from the locations where someone stopped, and most likely performed an activity, during the day. The most used digital biomarker for this group was the number of locations visited (n = 8). The third and fourth groups used geolocation data to infer actual behaviors and activities. Particularly, the third group looked at indications of regularity and routine in patient’s life, with location entropy (n = 4) that was the most adopted digital biomarker. Only 1 study (fourth group) tried to infer actual daily activities (eg, employment, shopping, sports, social activities, recreational activities) from the geolocation data.

**Table 3.** Geolocation concepts and derived digital biomarkers

Geolocation Concepts and Derived Digital Biomarkers		Data	Reference(s)
Concept	Digital Biomarker		
Mobility	Distance traveled	8 (44)	^56^ ^,^ ^58–60^ ^,^ ^62^ ^,^ ^63^ ^,^ ^65^ ^,^ ^67^
	Number of changes in GSM cell ids	4 (22)	^53–55^ ^,^ ^58^
	Standard deviation of distances	3 (17)	^60^ ^,^ ^63^ ^,^ ^65^
	Maximum displacement from primary location	2 (11)	^63^ ^,^ ^65^
	Total number of cells visited	1 (6)	^53^
	Mobility rate^a^	1 (6)	^53^
	Maximum distance between locations	1 (6)	^63^
	Maximum distance from home	1 (6)	^60^
Locations	Locations visited	8 (44)	^51^ ^,^ ^52^ ^,^ ^56^ ^,^ ^57^ ^,^ ^59–61^ ^,^ ^67^
	Time spent at prespecified locations	7 (39)	^59^ ^,^ ^60^ ^,^ ^62–65^ ^,^ ^68^
	Time spent outdoors	2 (11)	^66^ ^,^ ^67^
Regularity	Location entropy	4 (22)	^59^ ^,^ ^60^ ^,^ ^63^ ^,^ ^65^
	Locational routine index^b^	1 (6)	^60^ ^,^ ^63^
	Diurnal movements indexes^c^	1 (6)	^59^
Activities	Out-of-home activities^d^	1 (6)	^57^

Values are n (%).

a Calculated as number of changes in cell ids/total number of cells visited.

b Locational routine index over 7 days to quantify the degree of repetition in terms of places visited with respect to the time of day over a specific period of time.

c A measure of daily regularity quantified using the Lomb–Scargle periodogram to determine the power in frequencies with wavelengths around 24 hours.

d Inferring different types of daily activities (eg, employment, shopping, sports, social activities, recreational activities, other).

### Association with clinical concepts

Overall, 14 articles^53–60^^,^^63–68^ used geolocation-derived digital biomarkers to study different aspects of schizophrenia and bipolar disorder (see Table 4), with an association between digital and clinical variables shown in 12 cases. However, 7 of the 12 studies had a sample size below 20 and a follow-up time of shorter than 3 months. In some cases, associations were limited to individual patients, for example in case of rare outcomes.^60^^,^^65^ Only 1 study reported that some patients became upset and apprehensive because their whereabouts were being monitored.^62^

**Table 4.** Conditions and outcome measures found in the included studies

Clinical Concepts and Outcome Measures		Reference	Sample Size	Follow-Up	Was an Association Found With Geolocation-Derived Biomarkers?
Conditions	Outcome Measure				
Schizophrenia	Psychotic relapse (n = 2)	^65^	5	12 mo	Yes
		^60^	15	3 mo	Yes
	Symptoms (n = 1)	^63^	21	2-8.5 mo	Yes
	Self-reported daily activities (n = 1)	^57^	5	5 d	Yes
Bipolar disorder	Self-reported mood (n = 3)	^66^	6	6-8 wk	Yes
		^68^	7	12 wk	Yes
		^53^	6	6 mo	No
	Depressive state (n = 5)^a^	^67^	10	12 wk	Yes
		^54^	17	3 mo	Yes
		^55^	29	12 wk	Yes
		^58^	13	12 mo	No
		^59^	36	3-12 mo	Yes
	Manic state (n = 4)^b^	^67^	10	12 wk	Yes
		^58^	13	12 mo	Yes
		^55^	29	12 wk	Yes
		^54^	17	3 mo	No
	Social rhythm metric (n = 1)	^56^	7	4 wk	Yes
Schizophrenia and bipolar disorder	Violent behavior (n = 1)	^64^	27	7 d	No

a As measured as: Hamilton Depression Rating Scale (n = 4),^54^^,^^55^^,^^58^^,^^67^ Quick Inventory of Depressive Symptomatology–Self-Report (n = 1).^59^

b As measured as: Young Mania Rating Scale (n = 4),^55^^,^^58^^,^^67^^,^^68^ Mania Self-rating Scale (n = 1).^67^

#### Schizophrenia

Two articles^60^^,^^65^ explored the association between geolocation-derived digital biomarkers and psychotic relapse, in proximity of which they found changes in mobility and time spent at the primary location, respectively. However, these results have to be carefully interpreted due to the small sample size of these studies.

Wang et al^63^ followed up 21 patients recently discharged from hospital over a period ranging from 2 to 8.5 months. During the study, patients used the CrossCheck app to self-report positive (eg, calmness, social interaction, sleep, clarity of thought, hopefulness) and negative (eg, depression, stress, delusions, and suspiciousness) items, which were originally defined in Ben-Zeev et al^74^ by using the Positive and Negative Affect Schedule questionnaire.^75^ The CrossCheck app was also used to record geolocation and other passively sensed data (eg, accelerometer, voice and phone use). Using generalized estimating equations^76^ new places visited had a negative association with negative items. They also found that location entropy and time spent at primary location were in the top 10 most important features for predicting positive scores with gradient boosted regression trees, while maximum distance traveled between 2 location points and standard deviation of distances traveled were in the top 10 most important features to predict negative scores.

Difrancesco et al^57^ developed and evaluated an algorithm to infer daily activities relevant to social functioning (ie, home, working, shopping, sport activities, and social and recreational activities) based on geolocation data. This was tested in a cohort of 5 patients with schizophrenia monitored for 5 days. They found a recall of 0.7 (ie, 7 activities of each 10 reported by participants were retrieved by the algorithm) and a precision of 0.8 (ie, 8 activities were retrieved accurately of each 10 activities retrieved by the algorithm).

#### Bipolar disorder

With 8 studies, mood was the most investigated clinical concept in bipolar disorder, including self-reported mood^53^^,^^66^^,^^68^ and specific mood states.^54^^,^^55^^,^^58^^,^^59^^,^^67^

Three publications reported on the association between geolocation-derived digital biomarkers and self-reported mood.^53^^,^^66^^,^^68^ Sabatelli et al^68^ followed up 7 patients with the MONARCA app for 12 weeks, and found negative correlation between self-reported mood and time spent at the clinic. They also found that self-reported mood was positively correlated with time spent outdoors. Gruenerbl et al^66^ found statistically significant correlation between self-reported mood and percentage of time spent outdoors during the day, when following up 6 patients for 6-8 weeks. However, the correlation they found was difficult to interpret, as it was negative for half of the patients and positive for the other half. Finally, Frost et al^53^ monitored 6 patients for 6 months with the MONARCA app and performed an impact factor analysis that—based on correlation, information gain and statistical significance^77^—aimed at assessing the association between self-reported data (eg, sleep length, alcohol intake, medication adherence and activities) and smartphone sensed data (eg, geolocation, accelerometer data, smartphone activity) with self-reported mood. They found that mobility rate, calculated as the ratio between number of changes in GSM cells and the total number of different GSM cells visited, was placed 11th of all the 14 different covariates evaluated.

Another group of publications (n = 5)^54^^,^^55^^,^^58^^,^^59^^,^^67^ used geolocation-derived digital biomarkers to study specific mood states (ie, depressive or manic), which were derived by clinicians using different validated scores. In 3 publications,^54^^,^^55^^,^^58^ such validated scores were analyzed by using mixed effects models. Two of these publications reported on the MONARCA project, with 17^54^ and 29^55^ patients followed for 3 months. Faurholt-Jepsen et al^54^^,^^55^ found that the number of changes in GSM cells was negatively associated with depressive states and positively with manic states (as measured through the HDRS and YMRS). These associations were among the strongest (in terms of *P* value) in the smartphone-sensed data (ie, geolocation and smartphone activity data).^55^ Furthermore, Beiwinkel et al^58^ monitored 13 patients with bipolar disorder for 12 months with the SIMBA app, and, conversely to Faurholt-Jepsen et al,^54^^,^^55^ found negative association between distance traveled and manic state (according to YMRS). Gruenerbl et al,^67^ who followed 10 patients with bipolar disorder for 12 weeks, found values around 0.8 for recall, precision, and accuracy for a naïve Bayes classifier including only the geolocation-derived digital biomarkers (ie, ranging from distance traveled to time spent outside and locations visited) to distinguish between depressive and manic states, as assessed by clinicians using HDRS, Self-Rating Mania Scale, and YMRS. This was the highest performance among the smartphone-sensed data in the study. The last article looking at mood was by Palmius et al,^59^ who followed 22 patients and 14 healthy volunteers for 3-12 months. They were able to identify depressive states (as measured as the Quick Inventory of Depressive Symptomatology–Self-Report) with an accuracy of 0.85 by using different mobility and routine-related digital biomarkers in a quadratic discriminant analysis.

The last clinical concept evaluated in bipolar disorder was social rhythm, using the Social Rhythm Metric.^78^ Particularly, Abdullah et al^56^ used a support vector machine to model data from 7 patients with bipolar disorder who used the MoodRhythm app for 4 weeks. The support vector machine, which combined geolocation-derived data (ie, distance traveled and locations visited) and other smartphone sensed information (ie, accelerometer, audio and smartphone activity data) with self-reported mood, obtained a precision and recall of 0.85 and 0.86 in predicting stable or unstable Social Rhythm Metric score. Distance traveled and number of locations visited were the most important identified features.

#### Schizophrenia and bipolar disorder

Only Ben-Zeev et al^64^ included in their study both patients living with schizophrenia and bipolar disorder. They followed 27 hospitalized patients for 7 days with the CrossCheck app aiming to evaluate whether the time spent in different prespecified locations was associated with violent behavior. However, their nonlinear mixed effects analysis did not show any association.

## DISCUSSION

### Summary of findings

We performed a systematic review of the literature on the use of geolocation data to study SMI. We found only a modest number of relevant publications, mostly focusing on bipolar disorder. The digital biomarkers derived from the geolocation data aimed at assessing patient's mobility, locations, life regularity and daily activities. Locations visited, distance travelled, number of changes in GSM cell ids, and time spent in pre-specified locations were the most adopted geolocation-derived digital biomarkers. One study^62^ reported some patients becoming upset because of the continuous geolocation sensing. Twelve studies found some association between different SMI outcome measures and geolocation-derived digital biomarkers, but these have to be interpreted with caution because the majority of included studies were carried out for short periods of time and in small samples.

### Relation to other studies

Nicholas et al^79^ have previously published a systematic review of features and content quality of mobile apps for bipolar disorder, and Torous et al^80^ have reviewed the methodology and reporting of mobile health studies in schizophrenia. Our review is the first to explore digital SMI phenotypes derived from geolocation data. Similar to these 2 previous reviews, we found more studies on bipolar disorder than on schizophrenia.

Torous et al^80^ found poor reporting quality and a lack of adherence to the recently published World Health Organization mHealth Evaluation, Reporting and Assessment (mERA) guidelines.^81^ Similarly, studies in our review often did not provide information on sampling frequency or methods for processing geolocation. The mERA guidelines focus on quality and reproducibility of mobile health interventions rather than quality and reproducibility of digital phenotyping methods. We recommend that similar guidance were developed for digital phenotyping studies. This is becoming more important with the development of increasingly sophisticated sensors and technology embedded in smartphones. As has been done in other contexts (eg, systematic reviews with PRISMA),^82^ this guidance could be developed as an extension of the existing mERA guidelines.

Nicholas et al^79^ performed their search mid-2014 and did not identify apps that used smartphone sensors in bipolar disorder. In our review, the majority of included studies were published in the period 2014-2018, resulting in 9 included studies that employed the geolocation sensor, with 9 also capturing data from other sensors such as accelerometers. This exemplifies how quickly this field is emerging.

### What is the meaning of the findings and what are their implications?

Our study suggests that geolocation-derived digital biomarkers measuring patient mobility and daily locations have the potential to characterize mood in people with SMI. This is concordant with previous studies in other mental health conditions.^83–85^ Geolocation-derived biomarkers were also often more strongly associated with mood than digital biomarkers derived from other sensor data and patient-reported data. So, our findings warrant larger studies with longer follow-up times to strengthen the evidence for the association between geolocation-derived digital biomarkers and mood in people with SMI. This will help unlock the potential of smartphone-collected data to support tailoring of mental health services to patients’ needs, and informing timely interventions.^22^

To accelerate the uptake of digital biomarkers in health care and research settings, we make 2 recommendations. First, technology and intervention developers need to address the widespread privacy concerns related to continuously monitoring people’s whereabouts.^32^ This may include incorporating a functionality for patients to easily change the frequency of monitoring or turn it off completely at any time, or providing smartphone apps that facilitate processing of raw geolocation data on the device into (much less sensitive) metrics before sharing it with healthcare professionals or researchers. Second, looking at other fields, it seems that people are indeed willing to share their data, and especially location, if they expect to receive a clear benefit from it. For example, in applications that guide users in their journeys,^23^^,^^39^ there is a clear, direct advantage for the user of sharing data. This is the same in social media and location recommendation systems^24–27^ where people are willing to share their location data because the system is likely to suggest a place or activity that is useful for them. Therefore, we believe that digital health apps that collect these types of data should always aim to provide direct, tangible benefits to their users—even when their primary objective is a long-term goal such as prevention of psychotic relapse. For example, apps could give feedback to their users based on the collected data and associated risk assessments or analyses.

### Limitations

Our systematic review has 2 main limitations. First, digital phenotyping is a new field of research, for which terminology and MeSH (Medical Subject Headings) terms are still evolving. Therefore, although we used a comprehensive search strategy, we might have missed some studies. However, we do not anticipate that this limitation influenced our results nor our interpretation. Second, we have been as inclusive as possible in our systematic review, including different types of studies of varying quality. This led to inclusion of heterogeneous populations, making some findings difficult to generalize. It also led to inclusion of studies with small sample sizes and short follow-up times. The strength of the evidence for associations between the geolocation-derived digital biomarkers and clinical outcomes was therefore modest at best.

## CONCLUSIONS

Digital phenotyping of SMI using geolocation data is an emerging field, with 18 articles published to date. Although limited, the evidence on the association between SMI and geolocation-derived digital biomarkers shows potential for this approach to be used for continuous monitoring of patients in the community, especially to assess mood and related mental health states. In addition to improve their reporting of technical aspects, future studies should focus on other outcomes relevant to SMI (eg, mania, social functioning, social rhythm, positive symptoms such as hallucinations and delusions) that are currently underinvestigated. There is also a clear need for larger studies with longer follow-up times.

## FUNDING

This work was supported by the UK Engineering and Physical Sciences Research Council grant number EP/P010148/1 (The Wearable Clinic: connecting health, self and care) and by the National Institute of Health Research Manchester Biomedical Research Centre.

## AUTHOR CONTRIBUTIONS

PF, SNVdV, and NP conceived and designed the study. PF and AB were involved in data collection and synthesis. PF, AB, SNVdV, and NP drafted the manuscript. All authors critically revised the work for important intellectual content, and approved the final version of the manuscript for publication.

## Conflict of interest statement

None declared.
